# Relevance of Sympathetic Nervous System Activation in Obesity and Metabolic Syndrome

**DOI:** 10.1155/2015/341583

**Published:** 2015-04-30

**Authors:** Alicia A. Thorp, Markus P. Schlaich

**Affiliations:** ^1^Neurovascular Hypertension and Kidney Disease Laboratory, Baker IDI Heart and Diabetes Institute, Melbourne, VIC 3004, Australia; ^2^School of Public Health and Preventive Medicine, Monash University, Melbourne, VIC 3004, Australia; ^3^Department of Cardiovascular Medicine, Alfred Hospital, Melbourne, VIC 3004, Australia; ^4^Faculty of Medicine, Nursing and Health Sciences, Monash University, Melbourne, VIC 3800, Australia; ^5^School of Medicine and Pharmacology, Royal Perth Hospital Unit, Faculty of Medicine, Dentistry & Health Sciences, The University of Western Australia, Level 3, MRF Building, Rear 50 Murray Street, Perth, WA 6000, Australia

## Abstract

Sympathetic tone is well recognised as being implicit in cardiovascular control. It is less readily acknowledged that activation of the sympathetic nervous system is integral in energy homeostasis and can exert profound metabolic effects. Accumulating data from animal and human studies suggest that central sympathetic overactivity plays a pivotal role in the aetiology and complications of several metabolic conditions that can cluster to form the Metabolic Syndrome (MetS). Given the known augmented risk for type 2 diabetes, cardiovascular disease, and premature mortality associated with the MetS understanding the complex pathways underlying the metabolic derangements involved has become a priority. Many factors have been proposed to contribute to increased sympathetic nerve activity in metabolic abnormalities including obesity, impaired baroreflex sensitivity, hyperinsulinemia, and elevated adipokine levels. Furthermore there is mounting evidence to suggest that chronic sympathetic overactivity can potentiate two of the key metabolic alterations of the MetS, central obesity and insulin resistance. This review will discuss the regulatory role of the sympathetic nervous system in metabolic control and the proposed pathophysiology linking sympathetic overactivity to metabolic abnormalities. Pharmacological and device-based approaches that target central sympathetic drive will also be discussed as possible therapeutic options to improve metabolic control in at-risk patient cohorts.

## 1. Introduction

Activation of the sympathetic nervous system exerts a number of physiological responses either directly through stimulation of postganglionic sympathetic nerves localised in target organs or indirectly through the activation of powerful humoral systems. While the importance of sympathetic tone is readily acknowledged for cardiovascular and blood pressure regulation it is less well appreciated that activation of the sympathetic nervous system forms an integral part of energy homeostasis and can exert profound metabolic effects.

Insufficient physical activity and excess energy intake, coupled with genetic programming, have been attributed to the rising incidence of obesity, hypertension, dyslipidemia, insulin resistance, and hyperglycemia in western societies. More importantly, the clustering of these metabolic conditions, referred to as the Metabolic Syndrome (MetS) [1], has become increasingly more common with one-quarter of all adults predicted to have MetS. Given the augmented risk for type 2 diabetes, cardiovascular disease, and premature mortality associated with the MetS [2–4] there is growing interest in understanding the complex pathways underlying the metabolic derangements seen in the condition.

Accumulating data from animal and human studies suggest that central sympathetic activity plays a pivotal role in the aetiology and complications of MetS and its associated conditions. This is evidenced by a distinct preponderance for patients with the MetS to exhibit clinical signs of chronic sympathoexcitation such as elevated urinary noradrenaline levels, increased efferent muscle sympathetic nerve activity, and elevated rates of plasma noradrenaline spillover, even in the absence of hypertension [5–9]. Lifestyle modification is recommended as a first-line treatment of the MetS. Interestingly, recent evidence indicates that the benefits derived from diet and exercise induced weight loss are in part mediated by a reduction in sympathetic nerve activity [10]. Latest findings from clinical studies suggest pharmacological and device-based therapies (i.e., imidazoline agonists and catheter-based renal denervation) that specifically target central sympathetic outflow may also assist in improving metabolic control in these subjects. However, their long-term efficacy in ameliorating metabolic disease requires further investigation.

The purpose of this review is to present an overview of the evidence supporting the role of the sympathetic nervous system in metabolic control. This review will also focus on the possible mechanisms linking central sympathetic overactivity to the pathophysiology of the MetS, with particular emphasis on the putative role of the sympathetic nervous system in two key metabolic alterations, central obesity and insulin resistance. It will conclude by highlighting some of the emerging therapeutic options available for the treatment of MetS-related conditions that specifically target a reduction in central sympathetic activity.

## 2. Central Sympathetic Control and Its Role in Regulation of Metabolism

Central sympathetic outflow is principally driven by a network of neurons located in the rostral ventrolateral medulla. These neurons provide excitatory output to preganglionic neurons located in the intermediolateral cell column of the spinal cord that innervate several target organs through postganglionic sympathetic fibers. Excitatory drive can be intrinsically generated, chemically mediated, or differentially controlled via the cortical, limbic, and midbrain regions of the central nervous system. A number of afferent inputs from peripheral reflexes (i.e., arterial baroreflexes, chemoreceptors, and hormonal mediators) can stimulate the rostral ventrolateral medulla and alter sympathetic nerve activity via neurons that terminate in the nucleus tractus solitarius of the medulla oblongata. Additionally, circulating factors that are able to cross the blood-brain barrier via circumventricular organs can also influence central sympathetic outflow.

While the neuroanatomical interactions that govern the sympathetic nervous system are yet to be fully elucidated, sympathetic tone is recognised as an important mediator of cardiovascular function predominantly through its direct effects on beta-adrenergic receptors in the heart to modulate cardiac output and on alpha-adrenergic receptors in blood vessels to modulate arteriolar resistance and venous capacitance. Activation of adrenergic receptors in the kidney is also important for modulating blood pressure as neuronal noradrenaline promotes the release of renin from the juxtaglomerular apparatus, sodium retention, and vasoconstriction.

In terms of metabolism, the sympathetic nervous system is fundamental in controlling daily energy expenditure via the regulation of resting metabolic rate and initiation of thermogenesis in response to physiologically relevant stimuli, that is, changing energy states, food intake, carbohydrate consumption, hyperinsulinemia, and exposure to cold. Activation of sympathetic nerves innervating the liver, pancreas, skeletal muscle, and adipose tissue can also elicit acute catabolic responses (i.e., glycogenolysis and lipolysis). It is important to note that not all organs are targeted equally by the sympathetic nervous system with the metabolic effects ensuing from increased central sympathetic outflow dependent on the adrenergic receptors present in the target organ, the number of neurons recruited, and whether an individual is in a fasted or postprandial state.

**Figure 1:**
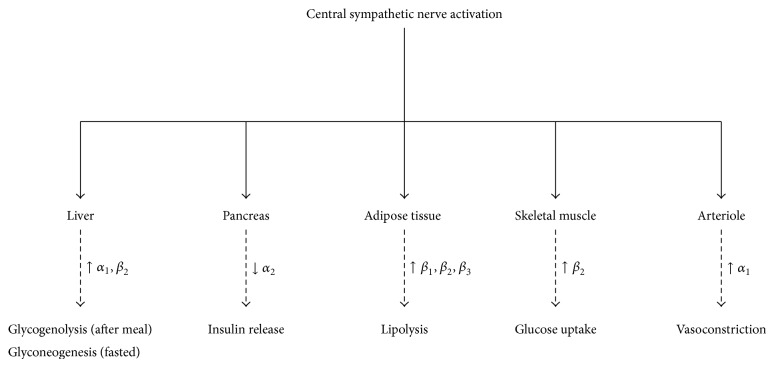
Role of the central sympathetic nervous system in metabolic control.

As shown in Figure 1, acute activation of splanchnic sympathetic nerves innervating parenchymal cells in the liver is shown to produce a rapid and marked production of glucose following a meal but promotes gluconeogenesis when fasted [11]. Activation of the adrenal medulla can also stimulate the release of catecholamines to further promote hepatic glucose production. In the pancreas, activation of splanchnic sympathetic nerves promotes glucagon secretion via beta-adrenergic receptors on islet cells, which is secondary to the inhibition of insulin secretion via alpha-adrenergic receptor activation. Sympathetic nerves innervating skeletal muscle can modulate glucose uptake independent of a concomitant increase in plasma insulin levels via activation of beta-adrenergic receptors using cAMP as the second messenger. Conversely, neuronal stimulation of alpha-adrenergic receptors in arterioles, which elicits vasoconstriction, impairs glucose uptake in skeletal muscle.

Compared to the liver, pancreas, and skeletal muscle, which are also under parasympathetic control, adipose tissue is only innervated by sympathetic nerves making it an important regulator of lipid mobilization. Central sympathetic outflow directly stimulates adipocyte lipolysis by binding to beta-adrenergic receptors in white adipose tissue to activate cAMP-dependent pathways to translocate inactive lipase. Equally, if alpha-adrenergic receptors are activated, lipolysis is inhibited. Noradrenaline has also been shown to decrease the uptake of triglyceride-rich lipoprotein into white adipose tissue via the inhibition of the rate limiting enzyme lipoprotein lipase (LPL), while beta-adrenergic activation in skeletal muscle stimulates LPL activity and promotes lipid uptake. Due to its ability to express various adipokines, white adipose tissue can also regulate sympathetic output through the production of centrally acting peptide hormones. Of those adipokines that are secreted from adipose tissue and modulate central sympathetic activity, leptin is the best described. It is able to cross the blood-brain barrier to act directly on leptin receptors in higher brain regions involved in the regulation of sympathetic tone. In brown adipose tissue, direct neuronal activation of beta-adrenergic receptors during exposure to different environmental stimuli (i.e., exposure to cold) is shown to promote thermogenesis via the uncoupling of mitochondria.

While the metabolic processes that ensue from acute sympathetic activation may be desirable under certain circumstances, it is clear that chronic stimulation of the sympathetic nervous system has the potential to augment risk for MetS, through the development of obesity, hyperglycaemia, insulin resistance, and hypertension.

## 3. Metabolic Syndrome, Prediabetes, and Diabetes Risk

The MetS is a constellation of metabolic abnormalities characterized by central (abdominal) obesity, insulin resistance, hyperglycemia, dyslipidemia, hypertension, and systemic inflammation that confers significant risk for the development of both type 2 diabetes and cardiovascular disease [1]. Central obesity is considered the cardinal feature of MetS with several major organizations recognizing waist circumference as the primary screening tool for MetS [12]. It is notable that subjects with central obesity often have more metabolic disorders and consequently are at greater risk of developing diabetes than those without central obesity [13]. Prediabetes is defined by impaired fasting glucose and/or impaired glucose tolerance with a glycosylated hemoglobin level (HbA1c) between 5.7 and 6.4% [14, 15]. Given that hyperglycemia is a component of the MetS and most individuals with MetS are insulin-resistant [16], it is often considered a prediabetic state. The observation that older, nondiabetic adults are two times more likely to have the MetS with hyperglycemia than hyperglycemia only confirms an overlap between the conditions [17].

The MetS is associated with a 5-fold increased risk for diabetes [18]. While most assume this to be driven by hyperglycemia, MetS without prediabetes carries a similar level of risk [19]. One of the reasons why the MetS is as strong a predictor of diabetes with or without prediabetes is the presence of central obesity [17] which not only acts to promote insulin resistance but can potentiate beta cell dysfunction through lipotoxicity [20].

## 4. Evidence of Sympathetic Dysfunction in Metabolic Conditions

There is substantial evidence in support of the sympathetic nervous system being exceedingly active in individuals with the MetS and its key metabolic alterations, central obesity, and insulin resistance. Compared to healthy lean individuals, obese adults are consistently shown to have raised urinary noradrenaline and metabolite levels [5] and elevated rates of whole-body noradrenaline spillover in plasma [21]. Furthermore, direct sympathetic nerve recording using microneurography demonstrates that obese individuals display increased resting sympathetic outflow to skeletal muscle [6, 22, 23]. Interestingly, the degree of sympathetic overactivity observed in obese individuals appears to be dependent on body fat distribution with central obesity shown to be associated with greater sympathetic nerve activation than subcutaneous obesity [7, 24]. The link between obesity and sympathetic overactivity is further strengthened by the observation that weight loss in obese individuals causes a marked decrease in muscle sympathetic nerve activity [6, 25] and increase in muscle sympathetic nerve activity following weight gain [26].

After a meal, the normal physiological response in healthy humans is to increase sympathetic nerve activity, as indicated by a rise in plasma noradrenaline concentrations and increase in muscle sympathetic nerve activity. Increased central sympathetic activity in the postprandial state is important to not only promote thermogenesis but induce compensatory peripheral vasoconstriction to maintain blood pressure following splanchnic vasodilatation [27]. Insulin-resistant individuals, particularly those whose insulin resistance is associated with central adiposity, display blunted sympathetic neuronal responses to physiological hyperinsulinemia, glucose consumption, and changes in energy states [8]. Indeed, Straznicky et al. confirmed that insulin-resistant subjects display a blunted sympathetic neural response to glucose ingestion compared with age- and blood pressure-matched insulin-sensitive subjects, despite a 2-fold greater increase in plasma insulin concentrations [28].

Evidence for sympathetic overactivity being linked to the onset of diabetes comes from several long-term prospective cohort studies that show both baseline noradrenaline levels and sympathetic reactivity predict future risk of insulin resistance and diabetes [29–31]. Experimental studies in patients with type 2 diabetes show significantly augmented resting muscle sympathetic nerve activity compared to individuals with prediabetes [32] or obesity [33]. Blunted sympathetic responsiveness to glucose and elevated arterial noradrenaline levels are also more exaggerated in treatment naive type 2 diabetics compared to individuals with impaired glucose tolerance [32] suggesting that the progression from prediabetes to diabetes is characterized by profound disturbances in central sympathetic nerve activity.

## 5. Sympathetic Overactivity as a Consequence of Metabolic Dysfunction

Several pathophysiological mechanisms linking central sympathetic overactivity with the MetS and its core components of central obesity and insulin resistance have been proposed [34] with ongoing debate as to whether sympathetic overactivity is a consequence or a cause of metabolic dysfunction.

Landsberg first proposed a link between the development of metabolic abnormalities and the sympathetic nervous system 25 years ago when he hypothesised that elevated circulating insulin levels, resulting from insulin resistance associated with obesity, caused an elevation in central sympathetic activity, which precipitated the development of hypertension [35]. His hypothesis was based on observations in rodents that showed that overfeeding leads to an increase in sympathetic nerve activity and rise in blood pressure. Subsequent studies in healthy adults confirmed this occurrence with the infusion of insulin, in the presence of stable glucose levels, shown to increase muscle sympathetic nerve activity independent of insulin's vasodilatory effects [36].

**Figure 2:**
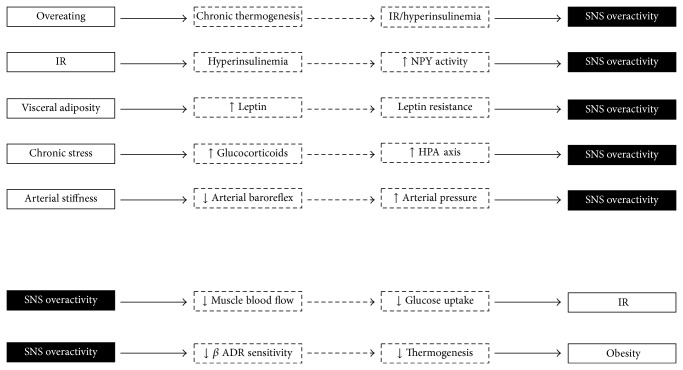
Schematic illustration of central sympathetic nerve overactivity as a consequence and cause of metabolic abnormalities. IR: insulin resistance; SNS: sympathetic nervous system.

Figure 2 highlights some of the proposed neurohumoral pathways that potentiate sympathetic overactivity. These include direct activation of the sympathetic nervous system by higher cerebral nuclei during overfeeding and disruption of hypothalamic insulin-signalling. Other indirect mechanisms (in the presence of obesity) include hyperinsulinemia, increased leptin and nonesterified fatty acids (NEFAs) release from excess visceral fat, and reduced baroreceptor sensitivity and activation of the hypothalamic-pituitary-adrenal axis. The proposed mechanisms for sympathetic overactivity as a distinct consequence of metabolic dysfunction are discussed.

### 5.1. Overeating

Animal and human studies clearly show that over- and underfeeding can modulate sympathetic nerve activity [37–39]. It has been proposed that chronic sympathetic activity in obesity is an adaptive physiological response used to stimulate thermogenesis and stabilize body weight during periods of overeating [40]. The notion that the autonomic nervous system acts to oppose weight change is supported by evidence from both normal weight and obese subjects that show modest weight gain is associated with an increase in sympathetic nerve activity and decrease in parasympathetic activity [38]. Chronic centrally mediated thermogenesis as a consequence of overfeeding does, however, come at the expense of sustained beta-adrenergic activation in the peripheral vasculature and kidneys which can precipitate a secondary rise in blood pressure via sodium retention and impaired pressure-natriuresis leading to obesity-related hypertension.

### 5.2. Hyperinsulinemia

The physiological association between insulin and sympathetic regulation is complex. In the body, insulin serves to reduce endogenous glucose production by directly inhibiting hepatic glycogenolysis or indirectly through the inhibition of lipolysis in adipose tissue, pancreatic glucagon production, or stimulation of insulin-dependent signalling pathways in the hypothalamus.

There are several lines of evidence to suggest that insulin exerts sympathoexcitatory effects via actions in the central nervous system [41]. Indeed, animal studies clearly show intracerebroventricular administration of insulin can augment central sympathetic activity [42]. Although insulin is not produced in significant amounts within the central nervous system, circulating insulin can gain access via saturable transport-mediated uptake across the blood-brain barrier [43, 44]. Cassaglia et al. recently identified the arcuate nucleus in the hypothalamus as one of the critical sites where insulin acts to increase sympathetic nerve activity and the sympathetic baroreflex [45]. It is postulated that the sympathoexcitatory effects of insulin result from suppressed inhibition of neuropeptide Y (NPY) neurons that project from the arcuate nucleus to the paraventricular nucleus. Following a meal, centrally acting insulin exerts anorexigenic effects in the hypothalamus via stimulation of proopiomelanocortin (POMC) and inhibition of NYP neurons. However, during chronic overfeeding, NPY gene expression in the arcuate nucleus is paradoxically elevated [46] as a consequence of hyperinsulinemia-mediated alterations in insulin receptor and insulin-signalling pathways potentiating “central insulin resistance.” Activation of NPY neurons promotes potent orexigenic effects via increased sympathetic outflow to the liver resulting in hepatic insulin resistance and increased endogenous glucose production. In animals, intracerebroventricular administration of NPY is shown to promote hyperphagia, hyperinsulinemia, insulin resistance, and obesity [47–49].

In humans, systemic but not local infusion of insulin stimulates an increase in sympathetic nerve activity supporting the notion that insulin's sympathoexcitatory effects are not mediated by a local mechanism [50]. Of note, the sympathetic response elicited by insulin in humans is far more heterogeneous than in animals. In healthy young adults, insulin infusion during constant plasma glucose concentration is shown to cause regionalised elevations in sympathetic nerve activity to skeletal muscle [51] but not the kidneys [52]. Furthermore the effect of acute hyperinsulinemia on muscle sympathetic nerve activity is more pronounced in lean adults compared to obese [36]. In hyperinsulinemic obese individuals with chronically elevated levels of circulating insulin, the normal central effects of insulin are blunted leading to increased endogenous glucose production (via activation of sympathetic outflow to the liver) and sustained sympathetic activation via the insulin feedback loop.

Given the observation that hyperinsulinemia causes regionalised sympathetic activity only (specifically to skeletal muscle) and subjects with obesity and hypertension are shown to have elevated renal noradrenaline spillover to plasma it is questionable whether hyperinsulinemia is the main mediator of central sympathetic overactivity observed in the MetS.

### 5.3. Increased Visceral Adiposity

Visceral white adipose tissue is a highly metabolic organ that not only accompanies but antedates other components of the MetS including insulin resistance, hypertension, hyperglycemia, and inflammation. Among the various indices of obesity, it is abdominal visceral obesity that is shown to be the strongest determinant of muscle sympathetic nerve activity in adults, with sympathetic nerve firing 55% greater in males with abdominal visceral obesity compared to men with subcutaneous obesity [24, 53].

Animal and human data show certain adipokines expressed by white adipose tissue, namely, leptin and nonesterified fatty acids, can contribute indirectly towards sympathetic nerve activity. Leptin is present in serum concentrations directly proportionate to adipose tissue mass and is shown to be elevated in human obesity [54]. It can act directly on skeletal muscle to impair glucose transporter-4 (GLUT-4) translocation and induce hyperinsulinemia resulting in coactivation of the sympathetic nervous system. Alternatively, leptin can act centrally in several brain regions (primarily the hypothalamus and brainstem) that control multiple metabolic functions via melanocortin-system-dependent pathways to increase sympathetic activity [55]. Very recent data suggests that leptin also acts at the level of the nucleus of the solitary tract to alter neurons involved in baroreflex sensitivity [56]. The main central effect of leptin is a reduction in appetite and increase in energy expenditure via sympathetic activation. It has been postulated that, in obesity, neurons located in the ventromedial hypothalamic nucleus that express leptin receptors become desensitized to chronically elevated levels of leptin (hyperleptinemia) suppressing its anorexigenic effects while selectively preserving sympathoexcitation (known as “selective leptin resistance”) [57, 58]. Animal models of obesity support this with leptin resistance in the hypothalamus shown to decrease satiety but preserve sympathetic activity [59].

While acute administration of leptin elicits a marked rise in muscle sympathetic activity in healthy lean men [60] there is some doubt as to whether leptin is the principal driver of chronic sympathetic activation in the MetS. Compared to nonobese adults, adults with high levels of subcutaneous fat who display 2-3-fold higher plasma leptin levels do not exhibit increased muscle sympathetic nerve activity [53]. Furthermore longitudinal studies in young Japanese adults reveal elevations in plasma noradrenaline levels precede both weight gain and increases in plasma leptin levels [29, 61] suggesting hyperleptinemia is ancillary to sympathetic stimulation associated with obesity.

Several studies in human show a positive correlation between circulating plasma nonesterified fatty acids (NEFAs), obesity, and insulin resistance [62]. As with leptin, NEFAs are able to act locally in peripheral tissue to disrupt insulin signaling and impair glucose uptake or can act centrally. Although it is unclear how NEFAs activate central sympathetic nerve activity, the infusion of NEFA is shown to increase muscle sympathetic activity in lean healthy adults [63]. There is also evidence that central sympathetic activation contributes to the increased alpha-adrenoceptor mediated pressor sensitivity observed with acute elevations in NEFA [64]. Despite evidence to support a possible interaction between NEFA and the sympathetic nervous system, others have shown no change in whole-body and renal noradrenaline spillover during NEFA infusion [65] highlighting the need for further research in this area.

### 5.4. Activation of the Hypothalamic-Pituitary-Adrenal Axis (HPA)

Stress-induced elevations in glucocorticoids are associated with profound metabolic abnormalities including insulin resistance, glucose intolerance, dyslipidemia, increased central adiposity, and hypertension suggesting that chronic stress may in part contribute to the development of the MetS [66]. Findings from a nested, case-control study of the Whitehall II study show both the hypothalamic-pituitary-adrenal HPA axis and sympathetic nerve activity are elevated in MetS subjects [67] providing a possible causal link between chronic stress and sympathetic activation. Experimental findings from animal and human studies lend further support to the coactivation of these brain centres during metabolic abnormalities, that is, obesity-induced hyperinsulinemia. Direct intracerebroventricular administration of corticotropin-releasing hormone (CRH) in primates is shown to promote sympathoexcitatory effects that lead to an increase in plasma noradrenaline levels, hyperglycemia, and elevations in blood pressure. Importantly, these effects are only attenuated following administration of a ganglionic blocker not a CRH antagonist [68] suggesting hyperglycemia associated with HPA axis activation is secondary to central sympathetic activation. In obese adults with increased sympathetic nerve activity, chronic administration of dexamethasone is shown to attenuate elevations in both plasma cortisol and muscle sympathetic nerve activity but not in lean adults [69].

### 5.5. Reduced Baroreflex Sensitivity

The arterial baroreflex is designed to buffer beat-to-beat fluctuations in arterial blood pressure. It exerts profound sympathoinhibitory effects during increasing arterial pressure [70]. In patients with MetS, the sympathoinhibition and sympathoexcitatory effects induced by baroreceptor stimulation and deactivation, respectively, are impaired [8]. Arterial baroreflex activity is also blunted in patients with visceral obesity [71], hypertension [72], insulin resistance, and early onset diabetes [28] suggesting baroreflex impairment may play a causal role in the sympathoexcitatory state observed in the MetS. Baroreceptors located in the carotid sinus and aortic arch are highly sensitive to stretch in the vessel wall and it has been postulated that reduced arterial distensibility can blunt baroreceptor discharge during increased arterial pressure resulting in chronic stimulation of efferent sympathetic outflow to the periphery.

## 6. Sympathetic Overactivity as a Cause of Metabolic Dysfunction

In support of a primary role of the sympathetic nervous system in metabolic abnormalities that cluster to form the MetS, several prospective studies clearly show that elevated noradrenaline levels can precede clinical manifestations of obesity and hypertension. In an elegant study of Japanese ship builders, Masuo et al. showed increased levels of circulating plasma noradrenaline independently predicted weight gain, elevated insulin levels, and blood pressure elevation during a 10-year follow-up period [29, 61]. Similar results over a 20-year follow-up period have been observed for weight gain in Norwegian men [73].

### 6.1. Obesity

Julius et al. first proposed that increased sympathetic activity was the primary defect leading to insulin resistance and weight gain in obese adults [74]. Increased sympathetic nerve activity is vital in the dissipation of energy following food consumption via activation of beta-receptors and it is proposed that chronic sympathetic nerve activity can potentiate weight gain leading to obesity as a consequence of diminished sensitivity of beta-adrenoceptors. In vitro and in vivo studies clearly show that prolonged adrenergic stimulation results in desensitization of beta-receptor mediated responses [75, 76]. Downregulation of beta-adrenoceptors leading to a blunted thermogenic response to food can potentiate insulin resistance and perpetuate the negative feedback cycle between insulin governing sympathetic outflows. Evidence in support of weight gain being directly related to decreased beta-adrenergic responsiveness comes from studies that show that both short-term [77] and chronic [78, 79] pharmacological blockade of beta-receptors leads to an increase in body weight.

### 6.2. Insulin Resistance

Due to the complex interactions between the sympathetic nervous system, hyperglycemia, hyperinsulinemia, metabolism, and insulin resistance it is difficult to define the primary insult that leads to metabolic dysfunction. As alluded to above, there is evidence in support of hyperinsulinemia promoting sympathetic nerve activity. It has also been proposed that insulin resistance is a secondary phenomenon precipitated by an increase in sympathetic tone [20, 80]. Evidence that sympathetic overactivity precedes the development of insulin resistance and prediabetes is supported by findings from a study of young Norwegian males where elevation in plasma norepinephrine during a cold pressor test was shown to predict hyperglycemia and impaired insulin sensitivity (as measured by HOMA-IR index) at 18-year follow-up [30].

Increased sympathetic outflow to skeletal muscle plays an important role in glucose metabolism primarily through eliciting reductions in skeletal muscle blood flow [81]. Indeed, an acute increase in sympathetic nerve activity has been shown to cause insulin resistance in the forearm of healthy adults [81]. Alpha-adrenergic vasoconstriction resulting from chronic sympathetic activity can blunt postprandial increases in skeletal muscle blood flow impairing glucose uptake and stimulating additional insulin production by the pancreas leading to insulin resistance [82]. In support of elevated sympathetic nerve activity promoting insulin resistance through peripheral vasoconstriction, administration of peripherally acting vasoactive agents has been shown to improve insulin sensitivity in obese hypertensive patients [83, 84].

## 7. Targeting Central Sympathetic Inhibition to Improve Metabolic Control

Given the important role sympathetic overactivity plays in metabolic abnormalities, inhibition of the sympathetic nervous system represents a logical and attractive therapeutic approach to treat the MetS and potentially reduce overall diabetes and CVD risk. It is possible to reduce central sympathetic activity through lifestyle modification, namely, energy-restricted diets and physical training [6, 10, 85] although recent evidence suggests pharmacological and device-based interventions targeting central sympathetic outflow are also likely to be of benefit.

### 7.1. Lifestyle Intervention

Evidence from the Diabetes Prevention Program and Oslo Diet and Exercise study clearly demonstrate the important metabolic benefits that can be derived from intensive lifestyle interventions [86, 87]. Whilst exercise training is shown to improve sympathoinhibition (preferentially to the kidneys) [88] and potentiate baroreflex sensitivity [25, 89] there is strong evidence to suggest that weight loss, in particular abdominal fat loss, is the most important determinant of sympathetic neural adaption to improve hemodynamic and metabolic parameters in adults with the MetS. In an elegant study by Straznicky et al., middle-aged subjects with the MetS who lost 9% of their body weight after undergoing a 12-week hypocaloric diet were shown to have significantly improved resting muscle sympathetic nerve activity, whole-body noradrenaline spillover, and whole-body insulin sensitivity. Fasting serum triglycerides and plasma leptin levels were also reduced. The addition of moderate-intensity physical activity may help augment the improvements observed in sympathoinhibition and metabolic outcomes [10].

### 7.2. Pharmacological Therapy

Pharmacological inhibition of sympathetic nerve activity to achieve sustained weight loss and improvements in insulin sensitivity, glucose tolerance, dyslipidemia, and hypertension is currently under intense investigation. Therapies targeting central sympathetic outflow, in particular, are thought to offer the greatest benefits as they may not only improve metabolic control but also help to regress end-organ damage.

Imidazoline I_1_ agonists act centrally at the level of the rostral ventrolateral medulla to inhibit sympathetic drive. Moxonidine is a second-generation imidazoline I_1_ agonist for the treatment of mild-to-moderate hypertension via inhibition of central sympathetic outflow. Moxonidine is also shown to exert beneficial effects on a diverse range of metabolic parameters including improvements in indices of glycaemic control, insulin sensitivity, dyslipidemia, and inflammation [90–93]. It also promotes weight loss by lowering leptin plasma levels in obese patients [94] and has been associated with improved renal function [95, 96], endothelial homeostatic mechanisms [97], and a reduction in left ventricular hypertrophy [91].

The capacity of moxonidine to improve several metabolic measures in addition to its effect on blood pressure makes it an attractive therapeutic option for the treatment of MetS and to reduce overall CVD risk. In a recent large, multinational, open-label 6-month uncontrolled observational study daily moxonidine use (either as a monotherapy or as an adjunct therapy) was shown to not only reduce blood pressure but also lower the CVD risk profile of MetS patients. This was achieved by improving, albeit modestly, several metabolic indices ranging from weight reduction of 2.1 kg, decrease of 0.6 mmol/L in triglycerides, and decline of 5 beats/minute in heart rate [93]. Larger studies with longer duration of follow-up are needed to confirm these promising findings.

### 7.3. Device-Based Therapeutics

Catheter-based renal denervation has emerged as a safe and effective therapy to lower central sympathetic nerve activity. Indeed, several studies show renal denervation lowers (by 50%) renal sympathetic nerve activity (as assessed by renal noradrenaline spillover) [98], whole-body sympathetic nerve activity, and muscle sympathetic nerve activity [99]. The therapy, which involves selective radiofrequency ablation of efferent and afferent sympathetic nerves in the renal artery lumen, is currently only recommended for use in patients with resistant hypertension to lower uncontrolled blood pressure. However, distinct benefits have been reported in relation to glucose metabolism [100]. Most recently, Mahfoud et al. reported for 37 drug-resistant hypertensive patients who underwent renal denervation, improvements in systolic and diastolic blood pressure were accompanied by significant reductions in fasting plasma glucose, insulin, and C-peptide as well as 2-hour postload glucose levels at 3-month follow-up. Insulin sensitivity as measured by the HOMA-IR and IS quicki index measures was also improved at 6-month follow-up. These beneficial metabolic alterations were not observed in the control group (*n* = 13) who continued their usual medication regime [100].

Further support of the beneficial effects on glucose metabolism following renal denervation has been reported in patients with polycystic ovary syndrome [101] and obstructive sleep apnea [102], two conditions that are characterised by multiple metabolic disturbances. In women with polycystic ovary syndrome, renal denervation was shown to lower fasting plasma glucose, improve insulin sensitivity (assessed by euglycaemic hyperinsulinemic clamp), and reduce both muscle sympathetic nerve activity and whole-body noradrenaline spillover at 3-month follow-up [101]. Importantly these metabolic effects were shown to occur in the absence of any changes in body weight. In a small study of 10 patients with obstructive sleep apnea, renal denervation was associated with improved 2-hour glucose levels during an oral glucose tolerance test and reduced HbA1c levels at 6 months following renal denervation [102].

## 8. Summary

The sympathetic nervous system plays a pivotal role in regulating metabolic control. While acute sympathetic activation may be desirable under certain circumstances, it is clear that chronic stimulation of the sympathetic nervous system has the potential to augment risk for the MetS through the development of obesity, hyperglycaemia, insulin resistance, and hypertension. While the exact mechanisms are yet to be fully elucidated, several lines of evidence suggest that sympathetic nervous system overactivity is important both in the initiation and the maintenance of metabolic abnormalities commonly seen in the MetS. Latest findings from pharmaceutical and device-based clinical trials are encouraging for targeting central sympathetic activity to improve metabolic control in patients with the MetS who are at heightened risk for diabetes and cardiovascular disease. From a research perspective, studies are needed to ascertain whether these latest therapies can ameliorate metabolic disease and attenuate future end-organ damage commonly associated with chronic sympathetic nerve activity.
